# Supplementary material for: The origin of enhanced O2+ production from photoionized CO2 clusters
Source: Commun Chem. 2022 Feb 4;5:16. doi: 10.1038/s42004-022-00629-z (PMC9814840; doi:10.1038/s42004-022-00629-z)
Supplement: Supplementary file 1 — Supplementary information [file 42004_2022_629_MOESM1_ESM.pdf]

Supporting Information: The origin of enhanced O_2^+ production from photoionized CO_2 clusters

Smita Ganguly,[†] Dario Barreiro-Lage,[‡] Noelle Walsh,[¶] Bart Oostenrijk,[†] Stacey L. Sorensen,[†] Sergio Díaz-Tendero,[*,‡,§,∥] and Mathieu Gisselbrecht[*,†]

†*Department of Physics, Lund University, Box 118, SE-221 00 Lund, Sweden*

‡*Departamento de Química - Módulo 13 - Universidad Autónoma de Madrid -28049 Madrid , Spain*

¶*MAXIV laboratory, Lund University, Box 118, SE-221 00 Lund, Sweden*

§*Condensed Matter Physics Center (IFIMAC), Universidad Autónoma de Madrid, 28049 Madrid, Spain*

∥*Institute for Advanced Research in Chemical Sciences (IAdChem), Universidad Autónoma de Madrid, 28049 Madrid, Spain*

E-mail: sergio.diaztendero@uam.es; mathieu.gisselbrecht@sljus.lu.se

Supplementary Note 1 – Cluster production : Γ^* formalism

The CO_2 clusters were produced using a conical nozzle with opening angle of 20°, nozzle diameter of 150 µm and nozzle temperature estimated to be −10 °C. The stagnation pressure of CO_2 gas behind the nozzle was varied from 0.2 to 0.82 bar. The size of the clusters in the beam after supersonic expansion into the vacuum chamber through the nozzle depends on the geometry and temperature of the nozzle, the properties of the molecule and the stagnation pressure of the gas. Hagena's condensation parameter Γ^* is a dimensionless parameter[1] that is used to estimate the mean cluster size N_{mean} at a given stagnation pressure. Supplementary Fig. 1 shows the calculated N_{mean} and Γ^* values for our experimental conditions.

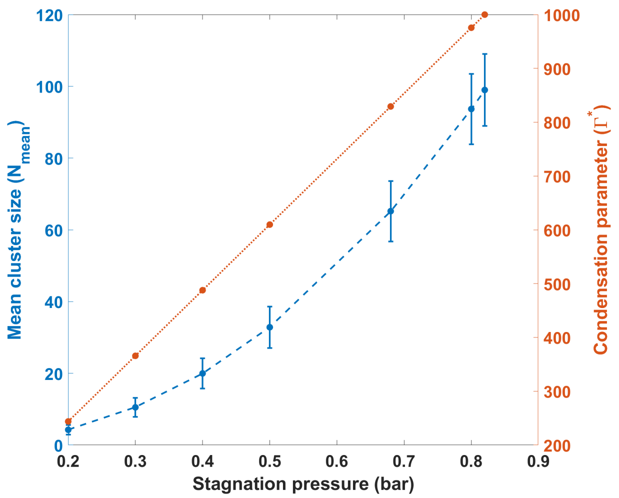

Supplementary Figure 1: Mean cluster size N_{mean} and condensation parameter Γ^* at the different experimental stagnation pressures used in this data set. The error bars for the N_{mean} values are calculated as described in Harnes et al.[2]

In the cluster beam, most of the molecules do not condense and hence among the measured photo-dissociation fragments, most of the signal comes from this molecular background. Quantitatively, we found that at $N_{mean} \sim 20$, only about 4% of the total double coincidence signal comes from the dissociation of cluster parent dications and the remaining 96% comes

from free molecular dissociation. Therefore, when comparing dissociation channels originating from free molecules and clusters we must consider the difference in population of the free molecules and clusters in the beam. Supplementary Table 1 shows the change in the total molecular and cluster signals detected for increasing mean cluster size. For events with one ion (C1) and events with two ions (C2) most of the signal detected is attributed to dissociation of the molecular background. As the mean cluster size increases, the cluster signal increases but for very large clusters the signal is limited by the detection efficiency. With an ideal spectrometer, the total cluster signal would increase as the mean cluster size and hence the population of clusters in the beam is increased.

Supplementary Table 1: Statistics of data attributed to molecular and cluster parent ions at different cluster sizes. C1 and C2 denote the events with single and double ion coincidences. The C1 cluster signal (%) includes the ions: O_2^+, $(CO_2)_n^+$ $(n = 1 - 10)$, $(CO_2)_mO^+$, $(CO_2)_mCO^+$ **and** $(CO_2)_mO_2^+$ $(m = 1 - 3)$. **The C2 cluster signal (%) includes the ion pairs:** $O_2^+/(CO_2)_n^+$, $O^+/(CO_2)_n^+$, $C^+/(CO_2)_n^+$, $CO^+/(CO_2)_n^+$ **and** $(CO_2)_m^+/(CO_2)_n^+$ **where** $(n, m = 1 - 10)$ **and** $m < n$.

Mean Cluster size	C1		C2	
	Molecular Signal (%)	Cluster Signal (%)	Molecular Signal (%)	Cluster Signal (%)
4	96.8	3.2	99.5	0.5
11	92.4	7.6	96.3	3.7
20	91.1	8.9	95.4	4.6
33	91.8	8.2	96.4	3.6
65	93.1	6.9	98.0	2.0
94	93.1	6.9	98.0	2.0
99	93.7	6.3	98.4	1.6

Supplementary Note 2 – Coincidence spectroscopy and Momentum imaging

A momentum-imaging spectrometer described in detail elsewhere[3,4] was used for experimental measurements. The ions were measured in the *true coincidence* regime with special care taken to have good alignment between the photon beam and the cluster beam. Additionally, to minimise false coincidences, we used low count rates with 50 µs acquisition time windows. The acquisition of each event is triggered by an electron emitted from the cluster/molecule after photoionisation. We assumed that the probability of k molecules being ionised within the acquisition time window t is a Poisson distribution with shape parameter λ,

$$f(k) = \frac{e^{-\lambda t}(\lambda t)^k}{k!} \quad \text{(Supplementary Equation 1)}$$

λ is measured as the electron count rate and when $\lambda \leq 7kHz$ the probability of measuring a false coincidence is estimated to be less than 5%. The events discussed in this paper are double-coincidence events where two ions were recorded within the 50 µs acquisition time window. These ions were produced by multiply charged parents. Cluster fragments larger than $(\mathrm{CO_2})^+_{10}$ were not observed in coincidence with other fragments, possibly due to the low detection efficiency of the heavy ions. We, therefore, acknowledge that the measurements have underestimated the yield of larger cluster fragments.

In general, if the probability of detecting an ion α^+ is $P(\alpha)$, then the probability of detecting n α^+ ions scales as $P(\alpha)^n$. For the $\mathrm{O_2^+}/(\mathrm{CO_2})^+_\mathrm{m}$ channel, the probability of detecting these two ions within the acquisition time window is given as $P(O_2^+) * P((CO_2)^+_m)$. As the value of $P((CO_2)^+_m)$ decreases for larger m,[5] our detection efficiency of the $\mathrm{O_2^+}/(\mathrm{CO_2})^+_\mathrm{m}$ channel also decreases for larger m.

Dalitz plot : Visualising momentum correlation of ions

For the ions measured in this experiment, we calculate the lab-frame initial momentum vectors and deduce information about the kinetics of the dissociation. Dalitz plots were used to visualize the momentum correlation between the ions detected in coincidence, the guide to read it is shown in Supplementary Fig. 2. For the O_2^+/CO_2^+ channel in the Dalitz plots, we identified two regions of interest (Regions A and B as indicated in Fig. 2 in main text). Momentum filtering of individual ions was used to isolate different events and each region was investigated independently in order to determine it's dependence on cluster size. The cluster size dependence of data points lying outside of the filtered Region A and B is not discussed.

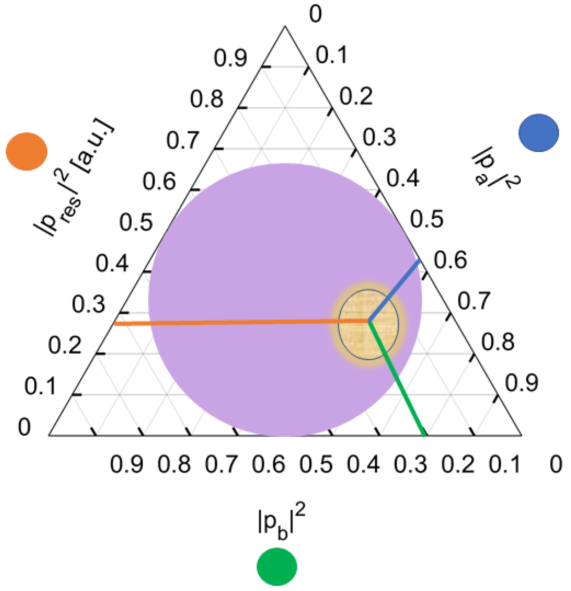

Supplementary Figure 2: Visual guide to interpret the Dalitz plot. The plot shows the momentum correlation between the particles a, b and the undetected particle with residual momentum. The sum of momentum vectors of three particles is assumed to be zero and the coordinates of each data point are given by $\epsilon_i = \frac{|\vec{P}_i|^2}{\sum_i |\vec{P}_i|^2}$ for each particle i. For selected point on the plot, $\epsilon_a = 0.56$, $\epsilon_b = 0.2$ and $\epsilon_{res} = 0.28$. The coordinates of all data points in the XY plane lie inside the purple circle due to conservation of momentum.

The Dalitz plot for the $O_2^+/(CO_2)_2^+$ and $O_2^+/(CO_2)_3^+$ channels are shown in Supplementary

Fig 3. The $O_2^+/(CO_2)_2^+$ channel shows a trend similar to the O_2^+/CO_2^+ channel in Fig. 2, a distribution appears to the right in the Dalitz plot as the cluster size increases. Region A for this channel is negligible. For the $O_2^+/(CO_2)_3^+$ channel statistics is not enough for reasonable interpretation and further analysis.

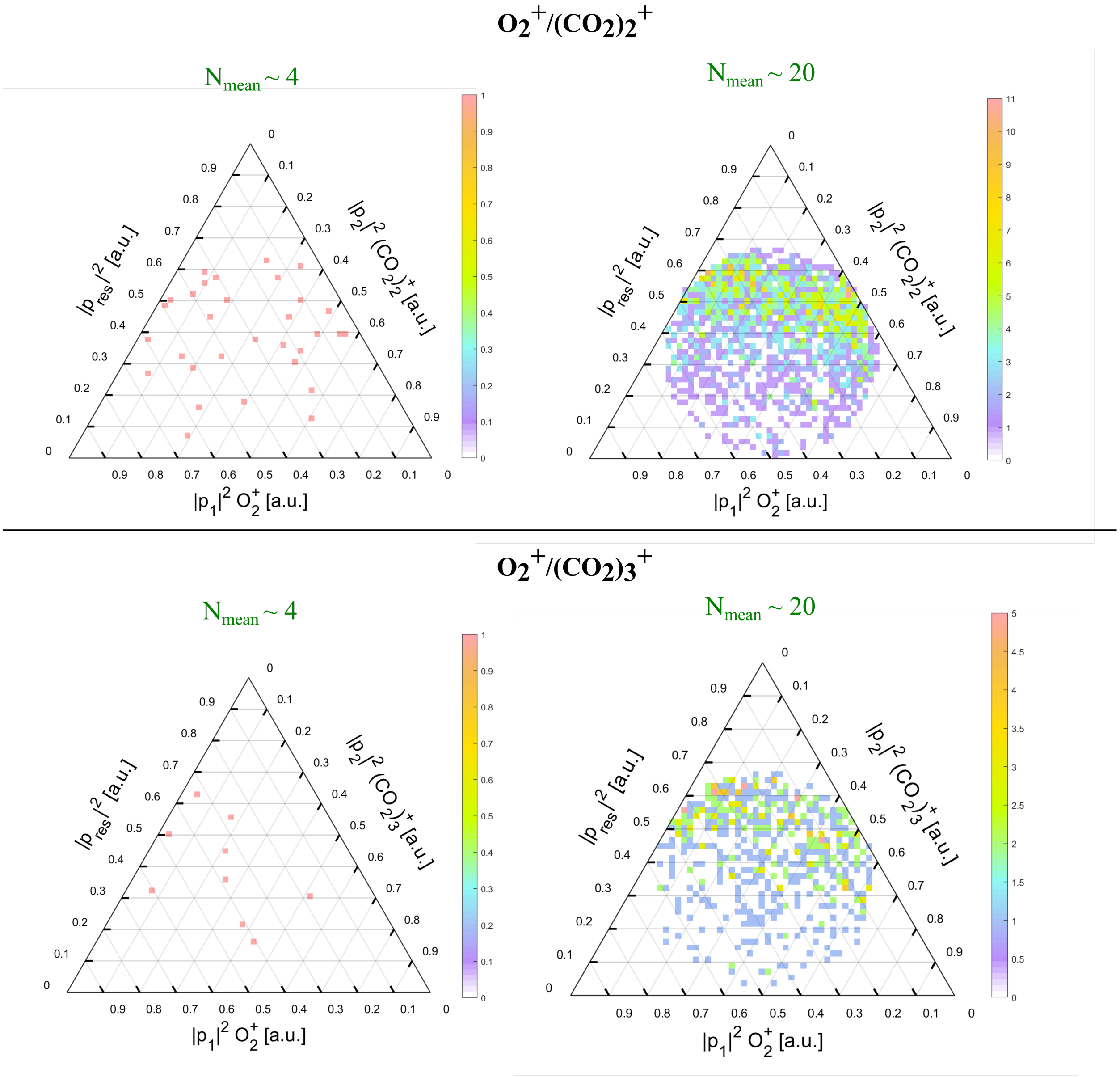

Supplementary Figure 3: The cluster size dependence of the Dalitz plots for the $O_2^+/(CO_2)_2^+$ and $O_2^+/(CO_2)_3^+$ channels.

The total yield of O_2^+ at different photon energies

In the article, we discussed the photo-dissociation mechanisms of CO_2 clusters ionised using soft X-rays at 320 eV and explored the cluster size dependence of O_2^+ production. Supplementary Fig. 4 shows the total yield of the all O_2^+ events from the cluster normalised w.r.t the O_2^+/C^+ channel from the CO_2 molecule for two separate data sets. Note that the ratio changes dramatically as a function of photon energy because the O_2^+/C^+ channel is extremely sensitive to the photon energy. Also, the background signal of the CO_2 molecules decreases slightly as the condensation parameter is increased to produces larger clusters. The yield also depends on the alignment between the cluster and photon beam. Overall, we observe that the CO_2 clusters are about 50-100 times more efficient in producing O_2^+ compared to the CO_2 molecule irrespective of photon energy.

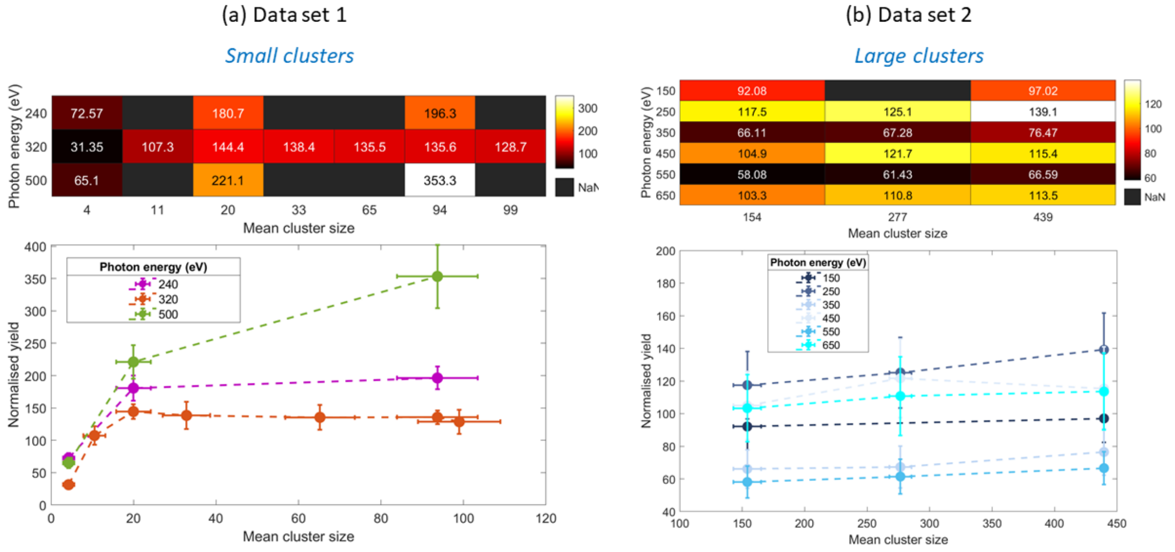

Supplementary Figure 4: The total yields for all O_2^+ events from cluster dissociation including single ion events as a function of increasing cluster sizes (4-440) at different photon energies. The yields are normalised to the O_2^+ yield from the molecule given by the coincidence signal O_2^+/C^+. (a) and (b) show the results for two separate data sets and demonstrate the variability of the yield. The CO_2 clusters were ionised using photon energies in the range 150 eV - 650 eV.

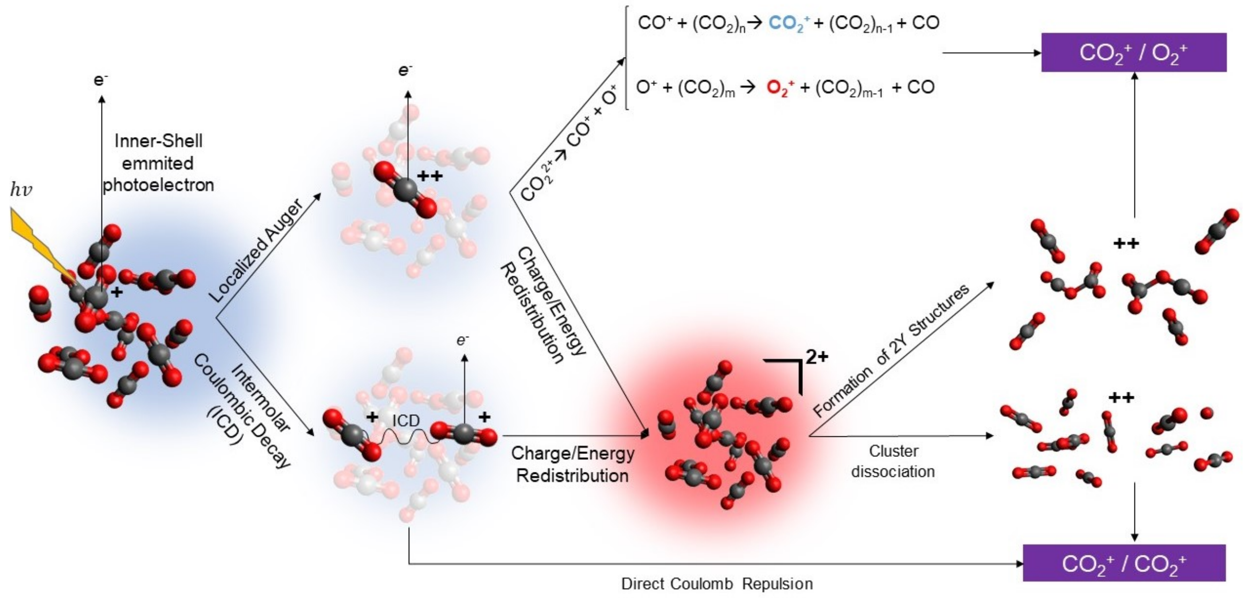

Supplementary Figure 5: Theoretical strategy followed for the study of doubly-ionized CO_2 clusters.

Supplementary Note 3 – '2Y structures'

Theoretical studies of the core-ionized CO_2 clusters were done for localised and delocalised Auger decay leading to different dicationic states as shown in Supplementary Fig. 5. When analyzing the stability of dicationic $(CO_2)_m^{2+}$ clusters, we identified an interesting type of covalently bound structure for those clusters composed of less than $m = 12$ CO_2 molecules. Supplementary Fig. 6 shows these structures for singlet and triplet electronic multiplicities, S = 1 and S = 3 respectively. All geometries were optimized with Density Functional Theory, using the M06-2X/6-31++G(d) level of theory. As can be seen in this figure, all clusters with size above $m = 4$ show the same core structure formed by 4 CO_2 molecules, resembling two opposing 'Y's. Hence the term 'double-Y structures' or '2Y structures'. For cluster sizes bigger than $m = 12$, we did not identify 2Y structures. We consider that this is due to the fact that the two positive charges are more easily redistributed along the cluster, therefore, resulting in a structure more closely resembling to the structure of the neutral cluster.

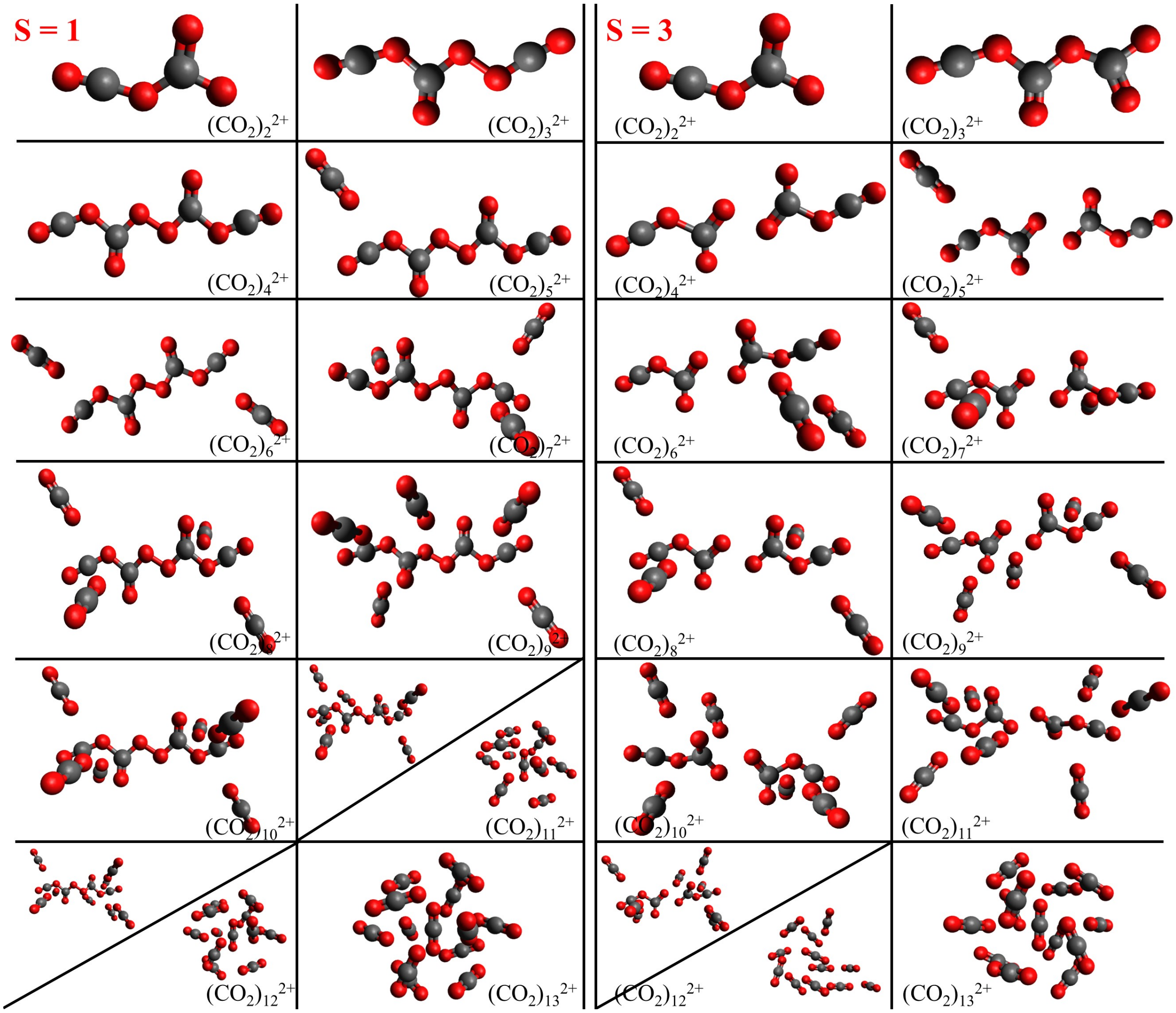

Supplementary Figure 6: Different structures for the dicationic $(CO_2)_m^{2+}$ clusters from $m = 2$ to 13. The two left columns represent the structures for the singlet electronic ground state S = 1, while the two right columns represent the structures for the triplet S = 3 state. Notice how the '2Y' structures disappear for cluster sizes $m > 12$.

Interestingly, the 2Y structures for the singlet S=1 show a shorter O-O bond distance (around 1.42 Å) while the same bond distance in the triplet S=3 is considerably longer (1.91 Å). This is also reflected in the calculated adiabatic ionization potentials.

Supplementary Fig. 7 contains two plots depicting the Adiabatic Ionization Potentials (AIPs) and the second Vertical Ionization Potentials (VIP2s) for both the singlet and triplet 2Y structures. The Adiabatic Ionization Potentials (AIPs) of the 2Y dicationic structures

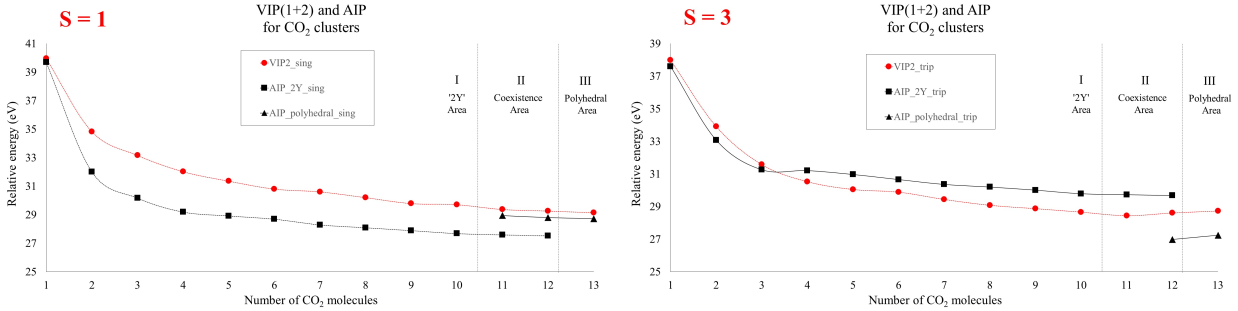

Supplementary Figure 7: Vertical and Adiabatic Ionization Potentials (VIP and AIP) of the CO_2 clusters with singlet S=1, and triplet S=3, spin multiplicity. In the AIP we highlight three different cluster size regions: (I) only structures of type 2Y are found, (II) polyhedral and 2Y structures coexist and (III) only polyhedral structures are found.

are significantly different for each of the two suggested multiplicities. Notice how the second Vertical Ionization Potential (VIP2) for the CO_2 clusters in the triplet S=3 state is lower in energy than the corresponding VIP2 in the singlet state. However, the AIPs for the singlet 2Y structures lie lower in energy, therefore their formation is a thermodynamically favourable process. In contrast, the AIPs for the triplet 2Y structures lie above both vertical ionization potentials.

For both multiplicities, the smallest $(CO_2)_m^{2+}$ clusters ($m < 12$) are observed to effectively redistribute the two extra charges without breaking (coulombic explosion) by creating covalently bonded 2Y structures. An atomic charge analysis for these structures has been analysed in the framework of the Mulliken population analysis. It is well known that the Mulliken results are very basis dependent and, therefore, inconsistencies may be obtained. In order to avoid them, we have also studied the atomic charges by employing the NBO (Natural Bond Orbital) method. Supplementary Fig. 8 shows the NBO charge analysis of $(CO_2)_4^{2+}$, $(CO_2)_8^{2+}$ and $(CO_2)_{13}^{2+}$. Notice that the two positive charges remain in the core 2Y structure, mainly on the two CO_2 molecules participating in the O–O bond; whilst, for the

larger cluster, the fifth and further CO_2 molecules act as neutral dipoles.

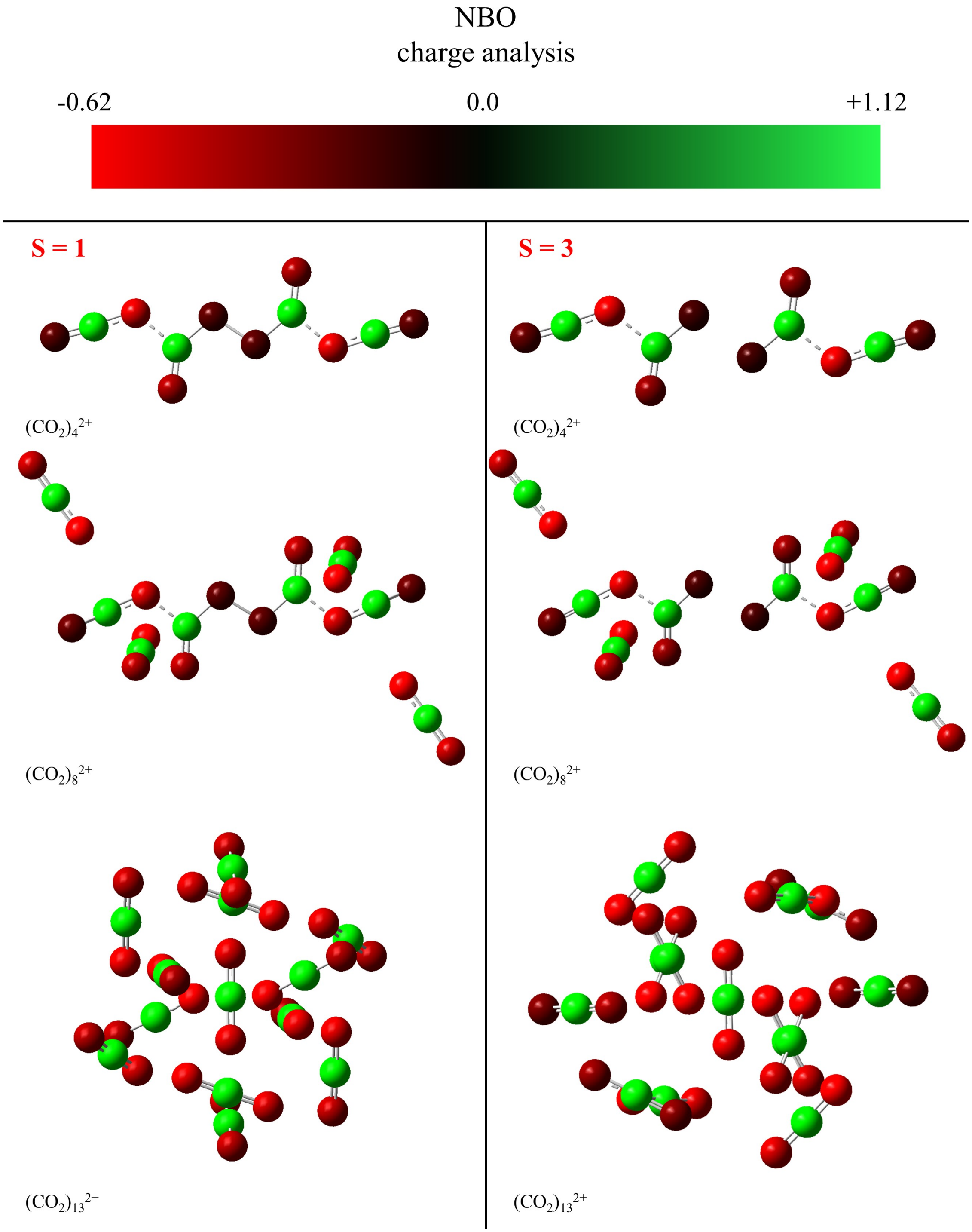

Supplementary Figure 8: NBO charge analysis for the $(CO_2)_4^{2+}$, $(CO_2)_8^{2+}$ and $(CO_2)_{13}^{2+}$ clusters with two different spin multiplicities: singlet S=1, and triplet S=3.

Supplementary Note 4 – Formation of CO_2^+/O_2^+ – Reaction pathways

Intermolecular Coulombic Decay

In this work, several mechanisms for the formation of the CO_2^+/O_2^+ fragmentation pathway have been proposed specifically for the most stable dicationic electronic state S=3.

We have focused on the tetramer, $(CO_2)_4$, due to the fact that it can form the characteristic 2Y structure. Once this cluster undergoes double ionization, the system can further evolve in a thermodynamic way by either coulombic explosion of the cluster, leading to the CO_2^+/CO_2^+ exit channel, or by stabilization in the 2Y $(CO_2)_4^{2+}$ structure. From here the system can evolve in two ways, either by a homolytic or a heterolytic cleavage.

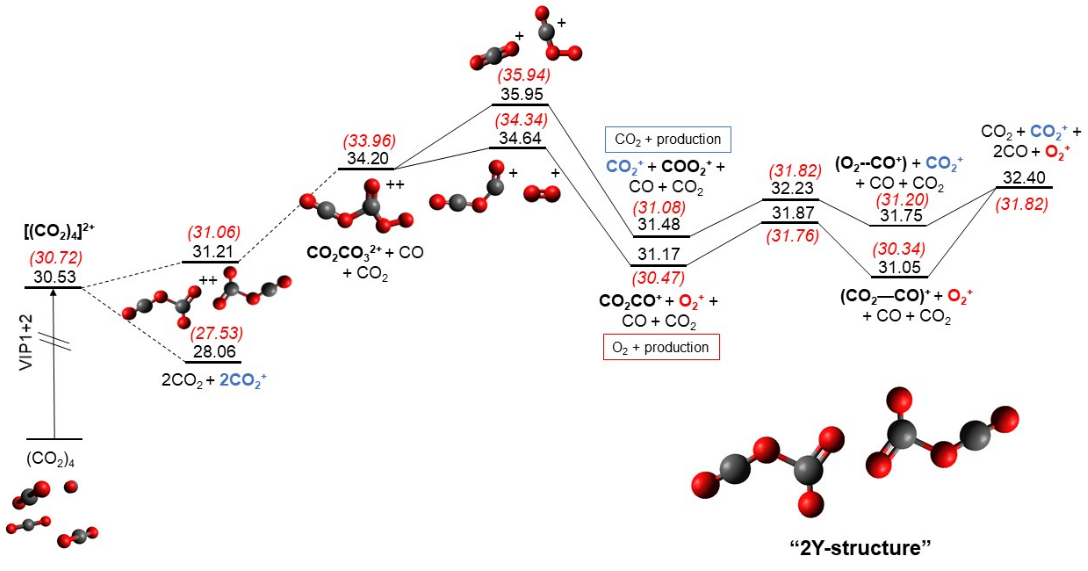

Supplementary Figure 9: Potential Energy Surface exploration for the evolution of the homolytic cleavage of $(CO_2)_4^{2+}$ after the formation of a 2Y structure. Relative energies (in eV) are referred to the neutral cluster. Relative energies in black text were calculated at a DFT-M062X/6-31++G(d) level of theory and corrected with the zero-point energy (ZPE). Energies in red text in brackets were computed as single-point energy calculations at the DLPNO-CCSD(T)/def2-TZVP level of theory over the geometries calculated at DFT level.

Supplementary Fig. 9 represents the homolytic cleavage of the $(CO_2)_4^{2+}$ 2Y structure. In this mechanism an intermediate, $CO_2CO_3^{2+}$, is formed with the release of neutral CO and CO_2. This doubly-charged fragment can also evolve in two different ways, one pathway has a higher energy barrier leading to the direct formation of CO_2^+ and the other is lower energy path leading to the formation of O_2^+. This reaction pathway was also calculated using a higher level of theory, DLPNO-CCSD(T)/def2-TZVP. The DFT results show a very good agreement with those computed using CCSD(T).Thus we can be confident on the DFT relative energies and using CCSD(T) is not required for the characterization of all the Potential Energy Surfaces.

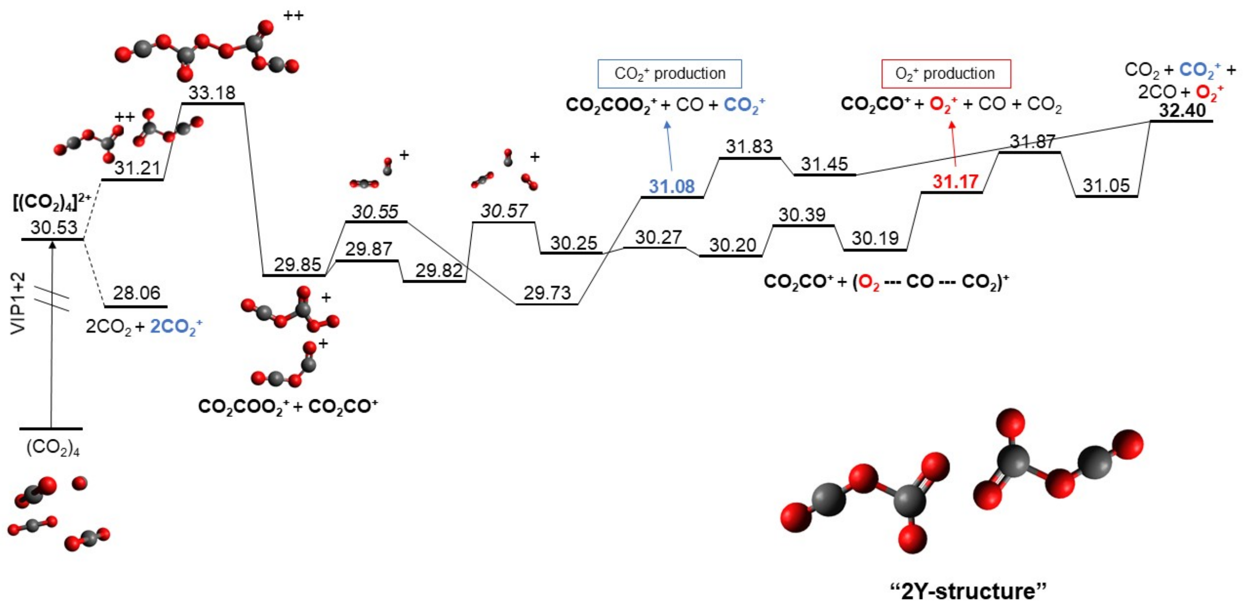

Supplementary Figure 10: Potential Energy Surface exploration for the evolution of the heterolytic cleavage of $(CO_2)_4^{2+}$ after the formation of a 2Y structure. Relative energies (in eV) are referred to the neutral cluster and corrected with the zero-point energy (ZPE).

On the other hand, Supplementary Fig. 10 shows the heterolytic cleavage of the $(CO_2)_4^{2+}$ 2Y structure. In this case two singly-charged molecules are produced; $CO_2CO_3^+$ and CO_2CO^+. Further fragmentation of each charged fragment barely differs in energy barriers. The

highest-energy transition state for each mechanism only differs by 0.02 eV (30.55 eV for CO_2CO^+ and 30.57 for $CO_2CO_3^+$). Therefore, the production of CO_2^+ and O_2^+ via this mechanism can simultaneously occur from the fragmentation of CO_2CO^+ and $CO_2CO_3^+$ respectively.

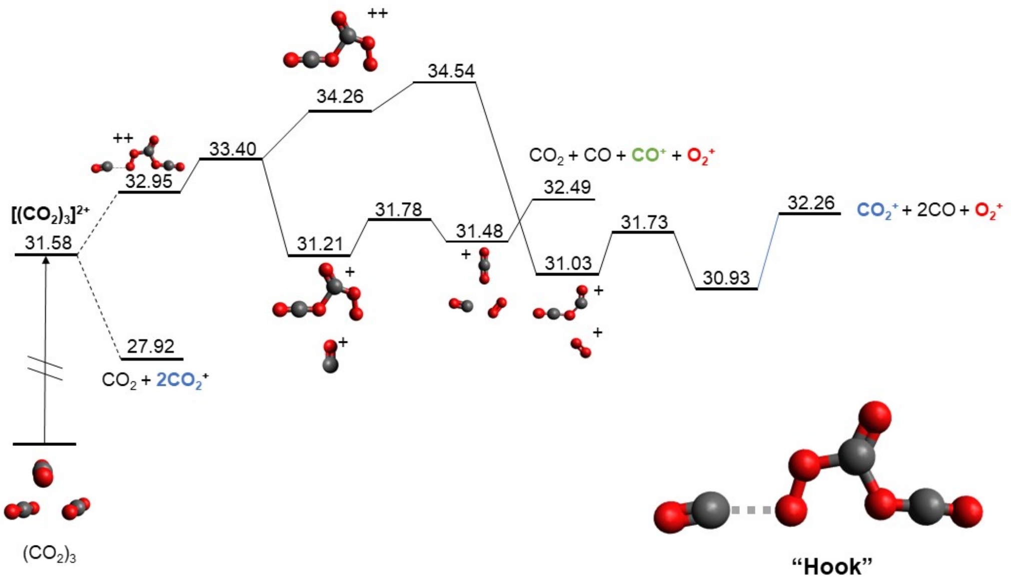

Supplementary Figure 11: Potential Energy Surface exploration for the evolution of the heterolytic and homolytic cleavage of $(CO_2)_3^{2+}$ after the formation of a 'hook'-like structure. Relative energies (in eV) are referred to the neutral cluster and corrected with the zero-point energy (ZPE).

Other cluster sizes, such as $(CO_2)_3^{2+}$, have also been considered, in contemplation of the possibility of other covalent dicationic species. In Supplementary Fig. 11 the evolution of a so-called 'hook' dicationic structure can again lead to a heterolytic and homolytic cleavage. The homolytic cleavage, is higher in energy and leads to the production of the desired CO_2^+/O_2^+ exit channel, while the heterolytic mechanism leads to the production of another interesting exit channel, CO^+/O_2^+, also observed in our experiments.

Localized Auger

A local Auger decay leads to the production of a doubly ionised single molecule, CO_2^{2+}, surrounded by several neutral CO_2 molecules. The excess positive charge facilitates the molecular Coulomb Explosion, leading to O^+ and CO^+. From the reorganisation of these fragments with neutral CO_2 clusters two separate mechanisms are defined. From the interaction of CO^+ with neutral CO_2 clusters, non-covalent interactions take place as a charge transfer mechanism creates CO_2^+. On the other hand O^+ interaction with neutral fragments results in the formation of $O(CO)_n^+$ type molecules along with a reorganisation that results in the emission of O_2^+. Supplementary Fig. 12 shows an example of these two mechanism starting with the reorganisation of O^+ and CO^+ with a $(CO_2)_7$ cluster. While the CO^+ molecule bonds in $CO(CO_2)_5^+$ in a non-covalent way, O^+ creates new covalent bonds with the formation of $O(CO_2)_2^+$. From $CO(CO_2)_5^+$, a charge transfer takes place from the CO to one of the CO_2 molecules in the cluster. At the same time $O(CO_2)_2^+$ evolves through several energy barriers with the eventual formation of O_2^+.

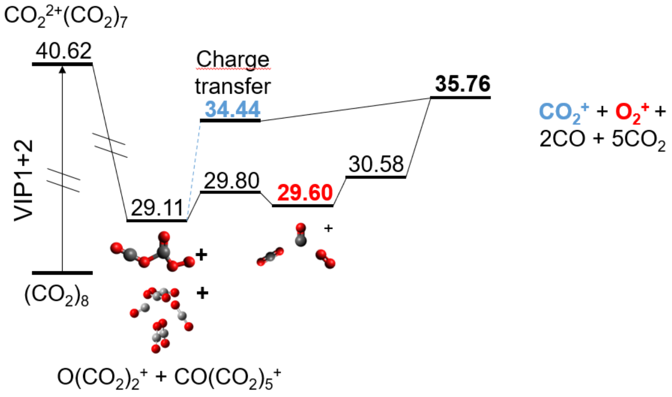

Supplementary Figure 12: Potential Energy Surface exploration for the formation of O_2^+ and CO_2^+ after a localized Auger process. Relative energies (in eV) are referred to the neutral cluster and corrected with the zero-point energy (ZPE).

Supplementary Note 5 – Dissociation energies (DE) and vertical ionization potentials (VIP)

As explained in the main article, the CO_2^+/O_2^+ exit channel, while interesting, is not the most populated one in the experiments. The channel CO_2^+/CO_2^+ is observed to be the most prevalent. The results of our theoretical calculations also indicate that the channel CO_2^+/CO_2^+ is the most favourable after ionization. These observations are also supported by considering the difference between the dissociation energy and the first + second vertical ionization potential, of the desired exit channel as shown in schematic in Supplementary Fig. 13 (a). Supplementary Fig. 13 (b) shows DE – VIP(1st+2nd) for 4 different channels. Those involving the formation of CO_2^+/CO_2^+ or $CO_2^+/(CO_2)_m^+$ lie below or are close to the Coulomb limit, while CO_2^+/O_2^+ and $(CO_2)_m^+/O_2^+$ lie above it, and, therefore, will require an excess of excitation energy to occur.

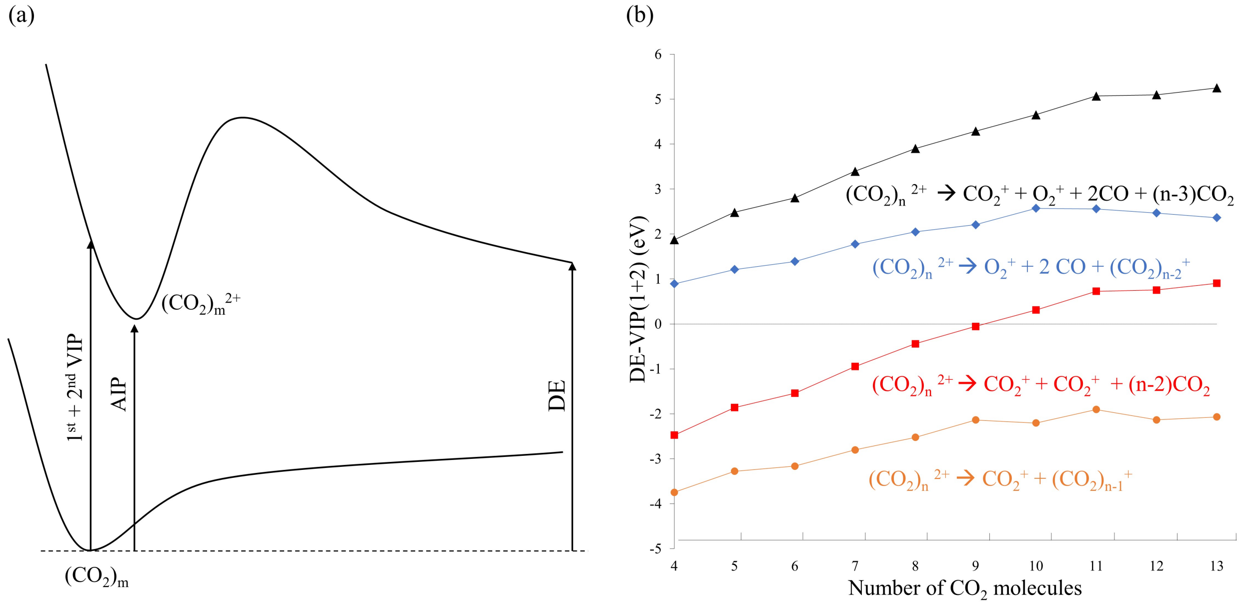

Supplementary Figure 13: (a) Scheme of the Vertical Ionization Potential (VIP) and Dissociation Energy (DE). (b) DE – VIP(1st+2nd) for different channels as a function of the cluster size for m = 1 to 13. The dashed line at DE – VIP(1st+2nd) = 0, is the Coulomb limit. If a channel lies below this limit this indicates that it will be energetically favourable.

Supplementary Note 6 – Statistics on the *ab initio* molecular dynamics

Ab initio molecular dynamics simulations were performed using the Atom-centered Density Matrix Propagation (ADMP) formalism.[6–8] Computational details are given in the section Methods in the main article. Here we present the statistics for the most relevant fragmentation channels. Supplementary Table 2 shows the percentage of trajectories where a $(CO_2)_5^{2+}$ cluster evolved toward channels CO_2^+/CO_2^+, CO_2^+/O_2^+ and CO_2^+/O^+, for three values of internal excitation energy $E_{exc} = 25$, 40 and 50 eV, and assuming the cluster in a singlet and in a triplet spin multiplicity. Our calculations were restricted to ground electronic state with singlet or triplet multiplicity, and at very specific excitation energy values, nevertheless, the same tendencies and a qualitative agreement is obtained from the comparison of the yield's ratio of the three channels between theory and experiments. The closest agreement between the simulations and experiment corresponds to the triplet spin multiplicity with excitation energy of 50 eV. The Auger spectra of a single CO_2 molecule shows the lowest energy peak centred at ~ 50 eV,[9,10] and we thus expect similar excitation energies for the clusters.

Supplementary Table 2: Statistics of the ADMP molecular dynamics simulations for $(CO_2)_5^{2+}$: Percentage of relevant channels and ratio between them. Ratio of experimentally measured yields for a cluster size $N_{mean} \sim 4$.

$(CO_2)_5^{2+}$ – Singlet – Theory			
Channel	25 eV	40 eV	50 eV
CO_2^+/CO_2^+	96.0%	80.3%	68.3%
CO_2^+/O_2^+	2.7%	11.7%	21.7%
CO_2^+/O^+	–	–	–
$[CO_2^+/O_2^+]/[CO_2^+/CO_2^+]$	–	0.15	0.32
$[CO_2^+/O^+]/[CO_2^+/CO_2^+]$	–	–	–
$(CO_2)_5^{2+}$ – Triplet – Theory			
Channel	25 eV	40 eV	50 eV
CO_2^+/CO_2^+	98.3%	70.0%	51.7%
CO_2^+/O_2^+	–	4.3%	9.0%
CO_2^+/O^+	0.7%	19.3%	25.3 %
$[CO_2^+/O_2^+]/[CO_2^+/CO_2^+]$	–	0.06	0.17
$[CO_2^+/O^+]/[CO_2^+/CO_2^+]$	0.007	0.28	0.49
$(CO_2)_4^{2+}$ – Experimental $N_{mean} \sim 4$			
$[CO_2^+/O_2^+]/[CO_2^+/CO_2^+]$	0.1416		
$[CO_2^+/O^+]/[CO_2^+/CO_2^+]$	1.026		

Supplementary References

(1) Hagena, O. F. Condensation in free jets: Comparison of rare gases and metals. *Zeitschrift für Physik D Atoms, Molecules and Clusters* **1987**, *4*, 291–299.

(2) Harnes, J.; Winkler, M.; Lindblad, A.; Sæthre, L.; Børve, K. Size of Free Neutral CO2 Clusters from Carbon 1s Ionization Energies. *The Journal of Physical Chemistry A* **2011**, *115*, 10408–10415.

(3) Laksman, J.; Céolin, D.; Månsson, E. P.; Sorensen, S. L.; Gisselbrecht, M. Development and characterization of a multiple-coincidence ion-momentum imaging spectrometer. *Rev. Sci. Instrum.* **2013**, *84*, 123113.

(4) Oostenrijk, B.; Barreiro, D.; Walsh, N.; Sankari, A.; Månsson, E. P.; Maclot, S.; Sorensen, S. L.; Díaz-Tendero, S.; Gisselbrecht, M. Fission of charged nano-hydrated

ammonia clusters – microscopic insights into the nucleation processes. *Phys. Chem. Chem. Phys.* **2019**, *21*, 25749–25762.

(5) Gilmore, I.; Seah, M. Ion detection efficiency in SIMS:: Dependencies on energy, mass and composition for microchannel plates used in mass spectrometry. *International Journal of Mass Spectrometry* **2000**, *202*, 217–229.

(6) Schlegel, H. B.; Millam, J. M.; Iyengar, S. S.; Voth, G. A.; Daniels, A. D.; Scuseria, G. E.; Frisch, M. J. Ab initio molecular dynamics: Propagating the density matrix with Gaussian orbitals. *The Journal of Chemical Physics* **2001**, *114*, 9758–9763.

(7) Iyengar, S. S.; Schlegel, H. B.; Millam, J. M.; A. Voth, G.; Scuseria, G. E.; Frisch, M. J. Ab initio molecular dynamics: Propagating the density matrix with Gaussian orbitals. II. Generalizations based on mass-weighting, idempotency, energy conservation and choice of initial conditions. *The Journal of Chemical Physics* **2001**, *115*, 10291–10302.

(8) Schlegel, H. B.; Iyengar, S. S.; Li, X.; Millam, J. M.; Voth, G. A.; Scuseria, G. E.; Frisch, M. J. Ab initio molecular dynamics: Propagating the density matrix with Gaussian orbitals. III. Comparison with Born–Oppenheimer dynamics. *The Journal of Chemical Physics* **2002**, *117*, 8694–8704.

(9) Kelber, J. A.; Jennison, D. R.; Rye, R. R. Analysis of the Auger spectra of CO and CO2. *The Journal of Chemical Physics* **1981**, *75*, 652–662.

(10) Püttner, R.; Sekushin, V.; Kaindl, G.; Liu, X.-J.; Fukuzawa, H.; Ueda, K.; Tanaka, T.; Hoshino, M.; Tanaka, H. A vibrationally resolved C 1s-1Auger spectrum of CO2. *Journal of Physics B: Atomic, Molecular and Optical Physics* **2008**, *41*, 045103.
